# Cone-Specific Filter-Based Neuromodulation: A Proposed Clinical Framework for Amblyopia, Strabismus, and ADHD

**DOI:** 10.3390/clinpract16010003

**Published:** 2025-12-25

**Authors:** Danjela Ibrahimi, José R. García-Martínez

**Affiliations:** 1 Facultad de Medicina, Universidad Autónoma de Querétaro, Santiago de Querétaro 76010, Mexico; danjela.ibrahimi@uaq.mx; 2 Brain Vision & Learning Center, Misión de Capistrano 117, Juriquilla, Santiago de Querétaro 76226, Mexico; 3 Maestría en Ciencias de la Ingeniería, Facultad de Ingeniería Mecánica y Eléctrica, Universidad Veracruzana, Poza Rica 93390, Mexico

**Keywords:** cone-specific neuromodulation, monochromatic and combined filters, amblyopia, strabismus, ADHD

## Abstract

Aim: To propose a standardized clinical protocol for cone-specific neuromodulation that classifies therapeutic filters for selective stimulation of S-, M-, and L-cones and translates optical and safety parameters into condition-specific frameworks for amblyopia, strabismus, and ADHD. Methods: Previously characterized spectral filters were re-evaluated using published transmittance and cone-excitation data to identify a reduced set of monochromatic and combined options with meaningful cone bias. These were integrated with α-opic metrology, international photobiological and flicker standards, and condition-specific neurophysiological evidence to define reproducible ranges for wavelength, corneal illuminance, exposure timing, temporal modulation, and safety verification. Results: The protocol consolidates eleven monochromatic and six combined filters into operational classes mapped onto mechanistic profiles for amblyopia, esotropia, exotropia, vertical deviations, and exploratory ADHD applications. All time frames and applications are presented as methodological anchors rather than efficacy claims. Conclusions: This work provides a structured, safety-anchored framework intended to guide protocol design and comparability in future cone-specific neuromodulation trials; therapeutic benefit must be demonstrated in prospective clinical studies.

## 1. Introduction

Despite decades of exploration, light-based interventions in vision science have remained fragmented and inconsistent, lacking a reproducible framework that can be translated reliably into clinical practice.

This work introduces a novel clinical protocol for cone-specific neuromodulation, emerging from our earlier studies that demonstrated light-induced cortical changes in strabismus and amblyopia, documented significant post-therapy improvements, identified the underlying visual evoked potential mechanisms (VEPs), and quantified filter properties to clarify their therapeutic potential. Building on this foundation, we now advance toward a standardized, photobiologically grounded framework that directly links cone pathways with clinical outcomes. In developing this protocol, we also integrate established knowledge on S-, M-, and L-cone physiology and key light parameters—spectrum, intensity, and timing—derived from the broader field of visual neuroscience. By aligning stimulus design with cone fundamentals, α-opic metrics, and validated elements of our prior work, we propose a coherent light-based therapy framework that connects retinal inputs to functional outcomes in real patients.

Against this backdrop, growing evidence has underscored the role of chromatic stimulation in influencing cortical activity and promoting functional recovery in patients with visual and neurodevelopmental disorders. Our previous studies initiated this line of research, providing the first systematic exploration of how cone-specific spectral filters influence visual and cortical responses. In our initial work, we demonstrated that baseline cortical activity differs significantly in patients with strabismus and amblyopia, and that selective spectral stimulation can modulate these differences [1]. Subsequent investigations showed that complete cycles of light therapy induced sustained cortical reorganization, with measurable improvements in binocular coordination and sensorimotor integration [2]. Independent work has also demonstrated that repetitive light stimulation can itself induce long-term cortical plasticity, reinforcing the evidence for light-based reorganization beyond our own findings [3]. Additional studies have consistently shown that selective chromatic stimulation elicits pathway-specific cortical and electrophysiological responses. Functional MRI and VEP investigations reveal that variations in cone-opponent contrast and hue generate distinct tuning patterns in human visual cortex [4,5,6]. Photoreceptor-directed paradigms further confirm that spectrally targeted stimuli evoke quantifiable cortical activation and perceptual effects [7], while comprehensive reviews highlight the physiological basis and clinical relevance of chromatic VEPs [8]. Further research employing VEPs confirmed differential cone contributions to cortical dynamics, while characterizing electrophysiological markers associated with S-, M-, and L-cone activation [9]. Most recently, a comprehensive analysis of spectral filter characteristics identified the optimal wavelength ranges and transmittance properties for clinical application, thereby providing the basis for the present protocol [10]. Collectively, these four studies provide the evidence base for the current work, which proposes a clinically applicable protocol for cone-specific visual therapy. Few studies to date have systematically integrated these elements into a therapeutic framework, and recent randomized controlled trial data further support the relevance of light-based interventions in improving visual outcomes in amblyopia [11].

However, despite such encouraging evidence, our own systematic evaluation of 33 therapeutic filters identified a critical barrier: the vast majority produced negligible visual-system activation, with cone stimulation often below 0.01% [10]. This inefficiency not only renders oversized filter inventories economically unsustainable but also undermines clinical reproducibility. The present protocol directly addresses this gap by refining the therapeutic toolkit to eleven rigorously validated monochromatic filters—each demonstrating consistent cone-selective efficacy—while designating six combined configurations to optional use given their lower impact. This streamlined framework eliminates redundancy, reduces costs, and, for the first time, establishes a reproducible and widely applicable foundation for cone-specific stimulation that can be reliably adopted across clinical centers.

To contextualize these findings, it is necessary to outline the neurophysiological basis of cone pathways and their cortical integration, which underpins the functional rationale for spectral stimulation.

S-cones, maximally responsive to shortwavelength light (∼420–440 nm), project predominantly to the koniocellular layers of the lateral geniculate nucleus (LGN), and subsequently to extrastriate cortical areas involved in motion, attention, and integrative visual processing [12,13]. M-cones (∼530–540 nm) and L-cones (∼560–565 nm) project mainly to the parvocellular pathway, providing high-resolution input to the primary visual cortex and influencing both dorsal and ventral streams [14,15]. The selective modulation of these cone pathways has been shown to impact visual perception and discomfort, with downstream relevance for attention and oculomotor function in specific conditions [12,16]. This cortical integration explains why cone-specific filters can elicit measurable changes in amblyopia and strabismus, where cortical suppression and imbalance of ocular dominance are key mechanisms [17].

Beyond strabismus and amblyopia, cone-specific stimulation has shown relevance in neurodevelopmental disorders. Studies in Attention-Deficit/Hyperactivity Disorder (ADHD) populations indicate altered temporal processing in visual and auditory domains, with evidence of reduced cortical activation in occipital and parietal regions [18,19,20]. Light-based interventions, particularly within specific wavelength bands, have been associated with improvements in attention, executive function, and sensory integration [21,22]. In Autism Spectrum Disorder (ASD), atypical early visual processing has been reported, and chromatic factors are implicated in visual comfort; broader light literature further supports physiological sensitivity to spectrum [23,24]. Recent work also emphasizes the contribution of circadian rhythm dysfunction to attentional variability in ADHD, strengthening the rationale for targeted light-based approaches [25]. Taken together, these converging lines of evidence bridge visual and neurodevelopmental findings into a unified therapeutic rationale, justifying the extension of cone-specific therapeutic applications to these populations.

Building on this translational basis, our protocol specifies spectral bands and α-opic targets aligned with these clinical needs, while also standardizing the critical light-delivery parameters—wavelength, intensity, exposure duration, and source. These parameters are consistently recognized as decisive for reproducibility and therapeutic efficacy across diverse light-based interventions. For example, photo-biomodulation studies highlight the biphasic dose–response and the importance of precisely defining wavelength, irradiance, and exposure time [26,27]. Clinical guidelines for light therapy in mood and circadian disorders emphasize standardized lux levels, exposure duration, and light-source characteristics [28]. Finally, systematic reviews and meta-analyses confirm that variations in intensity, spectral composition, and treatment timing critically influence outcomes [29].

Collectively, these findings demonstrate that cone-specific spectral filters constitute a promising neuromodulatory tool, capable of influencing both visual and cortical functions. By integrating our previous discoveries with the most recent evidence on wavelength specificity, light intensity, exposure duration, type of source, and clinical applications across visual and neurodevelopmental disorders, this paper provides a comprehensive introduction to a definitive clinical protocol. This proposal not only consolidates years of pioneering research but also addresses a critical gap in literature: the absence of a structured, evidence-based guide for the therapeutic use of spectral filters in visual rehabilitation. By consolidating prior discoveries into a reproducible, generalizable, and clinically grounded protocol, this work provides a foundation not only for immediate therapeutic application but also for the next generation of translational trials in visual and neurodevelopmental rehabilitation.

## 2. Methods

This section defines a reproducible methodological framework and outlines a standardized proposal for cone-specific neuromodulation. It integrates our prior experimental and clinical investigations with complementary evidence from the literature on light intensity, spectral composition, exposure duration, and delivery conditions (e.g., temporal modulation and measurement at the corneal plane). The references included here are not intended as a narrative review but as justification for parameter selection and safety verification, ensuring reproducibility across laboratories and future clinical studies. The protocol presented here is not a validated treatment guideline, but a framework grounded in quantitative spectral analysis and supported by converging clinical and neurophysiological findings from our previous work. The intention is not to impose a new therapeutic model, but to provide clear, verifiable standards for a type of filter-based neuromodulation that is already used empirically in some clinical settings without spectral validation or harmonized dosing criteria. By defining measurable optical and safety parameters, the framework offers a rational foundation for those clinicians who choose to employ such approaches, while enabling their systematic evaluation in future controlled trials. While therapeutic efficacy awaits formal clinical testing, this accumulated foundation provides a scientifically coherent and reproducible basis for forthcoming validation trials.

### 2.1. Study Design and Integration

The methodological framework is anchored in the anatomical and physiological properties of the S-, M-, and L-cone pathways, as well as in our prior experimental manipulations using monochromatic and combined filters. These data were translated into explicit operational parameters—spectral bands, corneal illuminance windows, block structures, and temporal modulation settings—under controlled photic conditions. The framework therefore functions as a translational bridge from basic cone neurophysiology and preliminary experiments to a reproducible set of specifications that can be independently implemented and audited for validation. Methodological development begins with study design and integration of prior evidence. Central to this framework are four peer-reviewed studies previously published by our group (Table 1). These works are not presented as new data but as independently verified datasets that quantified cone-specific optical transmittance, α-opic irradiance, and neurophysiological modulation through qEEG and VEP analysis. Their inclusion ensures methodological continuity and reproducibility, providing the empirical foundation upon which the present standardized proposal is built. Together, these datasets define the spectral, electrophysiological, and clinical rationale for the cone-specific neuromodulation framework and allow other laboratories to replicate or refine its parameters under comparable conditions.

#### 2.1.1. Experimental and Clinical Studies Providing the Basis for the Proposed Cone-Specific Neuromodulation Protocol

**Figure 1:**
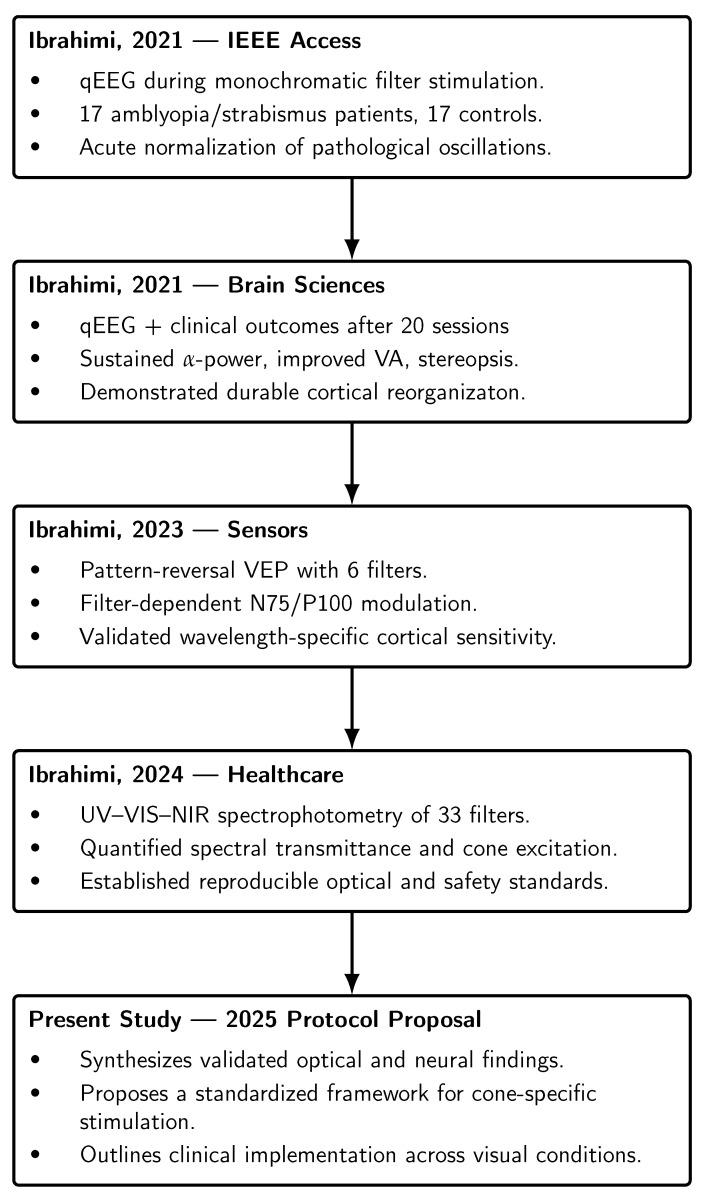
Sequential evidence chain linking the four prior experimental and clinical studies that form the foundation of the cone-specific neuromodulation protocol. Each study contributed a distinct layer of evidence—electrophysiological (qEEG, VEP), clinical (20-session neuromodulation), and optical/metrological (spectrophotometric characterization)—culminating in the present standardized clinical framework. The references in the figure are listed in the order of appearance as follows: [1,2,9,10].

Taken together, these studies delineate a sequential chain of evidence: acute normalization of pathological oscillatory activity [1], sustained cortical and functional reorganization with repeated stimulation [2], objective confirmation of filter-dependent modulation of early cortical responses [9], and quantitative metrology establishing reproducibility and safety [10]. This integrated foundation demonstrates that cone-specific neuromodulation is both physiologically effective and technically feasible, providing the direct rationale for its integration into a standardized clinical protocol. Figure 1 was added to visually summarizes the four core studies underpinning the present protocol, showing how each contributes a distinct layer of evidence—from electrophysiological validation to optical metrology—culminating in the proposed standardized clinical framework. This addition allows readers to appreciate the methodological continuity and scientific foundation without consulting all prior publications individually.

#### 2.1.2. Protocol Rationale

Building on this foundation, the protocol consolidates three strands of research: optical characterization of filters, electrophysiological validation of their cortical effects, and clinical assessment of functional outcomes. The rationale is to formalize these elements into a standardized methodological framework that specifies spectral ranges, illuminance, exposure timing, and safety safeguards. By linking cone-specific stimulation directly to known neurophysiological mechanisms and clinically measurable endpoints, the protocol ensures methodological reproducibility, translational relevance, and feasibility for implementation.

The next step is to ground these methodological choices in the neurophysiological architecture of S-, M-, and L-cone pathways, which define the logic for targeted stimulation.

### 2.2. Neurophysiological Foundations

The neurophysiological basis of this protocol is outlined below, describing the distinct pathways and cortical functions associated with S-, M-, and L-cones. These mechanisms form the rationale for specifying cone-targeted stimulation parameters in the proposed methodological framework.

#### 2.2.1. S-Cone Pathways

S-cones, maximally sensitive near 420–440 nm [30], project predominantly via koniocellular layers of the lateral geniculate nucleus into blob domains of primary visual cortex (V1), with subsequent relays to extrastriate regions including secondary visual cortex (V2) and color-biased sectors of visual area 4 (V4) [31,32,33]. Intrinsically photosensitive retinal ganglion cells (ipRGCs) integrate cone input, including S-cone–opponent signals, and project to nuclei mediating non-visual responses such as the suprachiasmatic nucleus and the olivary pretectal nucleus, thereby contributing to circadian entrainment and the pupillary light reflex [34,35,36,37]. Functionally, S-cone circuits establish blue–yellow opponency in early visual pathways [38] and can antagonize melanopsin responses in human pupil control, indicating that S-cone isolation should not be considered a proxy for circadian stimulation or alertness [39].

#### 2.2.2. M-Cone Pathways

M-cones, with peak sensitivity near 530–540 nm, contribute to the parvocellular red–green opponent pathway and, together with L-cones, to the luminance channel that drives magnocellular responses [30,40,41]. These inputs terminate in V1 layer 4C, where parvocellular and magnocellular signals remain partly segregated, and subsequently feed dorsal-stream regions including middle temporal visual area (MT/V5) and medial superior temporal area (MST) [33,42]. Functionally, these channels support motion sensitivity and contribute to binocular coordination and visuomotor control, although their specific role in pursuit–saccade coupling remains less directly demonstrated. While M-biased pathways are considered important for vergence and visuomotor stability, there is no direct therapeutic evidence from paradigms isolating M-cone input.

#### 2.2.3. L-Cone Pathways

L-cones, with peak sensitivity near 560–565 nm, predominantly feed the parvocellular pathway, providing high-resolution foveal input to V1 and projecting onward into ventral-stream regions such as V2 and V4 [30,33,43,44]. L-cone–biased signals underpin central visual acuity, fine spatial discrimination, and red–green opponency, integrated with combined L+M luminance contributions that support stable fixation and visual detail [13,45]. Clinically, L-biased stimulation has been proposed to aid central fixation and reinforce chromatic opponency in conditions such as amblyopia or exotropia, but causal evidence for direct feedback to higher oculomotor centers remains limited.

#### 2.2.4. Proposal: Cone-Targeted Stimulation Blueprint and Implementation Standards

Building on Section 2.2.1, Section 2.2.2 and Section 2.2.3, we propose a blueprint that translates cone pathway mechanisms into measurement-traceable operational parameters. The goal is to establish a framework that is selective, safe, and reproducible across laboratories, providing a methodological basis that can be adopted in both research and clinical contexts.

This rationale rests on two pillars: alignment with international standards and reproducibility across settings. The framework organizes S-, M-, and L-cone–weighted stimulation blocks by spectral band, corneal illuminance, exposure timing, temporal modulation, and photobiological safeguards (Table 2). Dose computation and safety are anchored in α-opic metrology and photobiological/flicker standards [46,47,48,49], alongside integrative guidance on non-visual responses [50,51]. Spectral tuning follows modern cone fundamentals [30] and circadian/α-opic metrology [52,53]. To ensure reproducibility, we recommend spectrally narrowband LEDs with calibrated spectroradiometric verification, since broad or composite sources may drift with dimming method, drive current, temperature, or operating time [53,54,55].

Parameters in Table 2 were proposed to reflect cone adaptation dynamics—shorter blocks for S-cones to counter rapid chromatic adaptation, and longer exposures for M- and L-cones to support visuomotor and foveal stability—while remaining within validated safety margins. Corneal illuminance windows were chosen to ensure physiologically effective α-opic stimulation without exceeding retinal hazard thresholds. Together, these values provide a reproducible framework that others may adopt for selective cone engagement. Full operational rationale and safety considerations are detailed in Sections Proposal: Measurement and Source Verification–Proposal: Safety Guardrails and Scope.

The operational dose windows, block designs, and safety constraints summarized here consolidate international standards and prior literature. These parameters represent scientifically derived implementation standards, proposed as a reliable foundation for testing cone-targeted neuromodulation in clinical research. Our contribution lies in the classification of eleven monochromatic and six combined filters and their mapping to disorder-specific protocols.

##### Proposal: Measurement and Source Verification

We propose that dose be defined at the corneal plane and reported as both photopic illuminance (lux) and α-opic equivalent daylight illuminances (EDIs) for cones, rods, and melanopsin [46]. Measurements should be taken with a calibrated lux meter and spectroradiometer, with a diffuser for uniform fields, periodic spectral/α-opic verification, and documented geometry (distance, field size, diffuser, orientation). Narrowband LEDs centered at 440, 530, and 570 nm, aligned with cone fundamentals [30], are recommended and should be spectrally verified before use. This procedure can be reproduced in clinical practice using standard, commercially available instruments, ensuring both reliability and transferability.

Beyond the cone-aligned narrowband sources, the framework assumes the use of broadband, low-flicker LED sources with controlled spectral output, adjustable illuminance, and stable correlated color temperature (≈5500 K), as recommended by international lighting and photobiological safety standards (CIE S 026:2018; ISO/CIE 8995-1:2025; IEEE 1789-2015; IEC 62471:2022). These specifications are provided as methodological requirements for future device construction and calibration, rather than as data obtained from a specific source.

Age-dependent adjustments are necessary: larger pupils in children increase retinal dose, while lens yellowing and smaller pupils in older adults reduce short-wavelength transmission [56,57,58]. Broad-spectrum sources (high-CRI white, RGB, RGW) are excluded unless closed-loop spectral feedback stabilizes α-opic output, as their spectra drift with dimming, drive current, temperature, and aging [59,60]. Target illuminances should follow workplace norms [61,62] to maximize ecological validity and comfort. All sessions are to be performed under controlled ambient lighting (<100 lx) to maintain photopic adaptation state and minimize pupil/mesopic variability, ensuring consistent retinal illuminance.

##### Proposal: Temporal Design and Block Structure

We propose that block durations be aligned with cone adaptation kinetics (Table 2). S-cone blocks remain brief (≈2 min × 4, ≈8 min total at 250–350 lx) to counter rapid chromatic adaptation (t½ ≈ 20 s; near-asymptote ≈1 min) and glare [30,63,64,65]. M-cone blocks are moderate (≈4 min × 4, ≈16 min at 400–500 lx), reflecting integration of magnocellular and parvocellular pathways [33,41,42]. L-cone blocks are longer (≈5 min × 4, ≈20 min at 500–650 lx) to sustain foveal fixation and object recognition [13,43,45]. Rest intervals (30–60 s) are included, and L-cone sequences use a ramp-up for comfort. Longer steady-state visual evoked potential (SSVEP) runs are feasible with breaks [66].

Illumination should follow strict modulation safety: delivered as DC or high-frequency PWM (≥1 kHz) in line with IEEE 1789-2015. Frequencies between 15–25 Hz are excluded due to seizure risk [67], and heterochromatic 32–40 Hz flicker is permitted only in controlled research at low modulation depth [68,69,70,71].

##### Proposal: Timing and Circadian Hygiene

We propose that scheduling follow circadian logic: S-cone blocks in the morning or early day, M- and L-cone blocks during daytime, and evening exposure avoided. Guardrails specify daytime melanopic EDI ≥ 250 lx and evening melanopic EDI ≤ 10 lx [52,53]. S-cone stimulation should be interpreted as a visual–neurophysiological input, not a circadian proxy, since S-cone pathways can oppose melanopsin in pupil control [39]. Standardized α-opic reporting is recommended to ensure reproducibility across laboratories [50].

##### Proposal: Safety Guardrails and Scope

We propose that participants be pre-screened for photosensitive epilepsy, migraine, severe photophobia, concussion, or pattern sensitivity [72]. Safeguards should include diffuser use, gradual ramp-up/down, emergency stop mechanisms, and active symptom monitoring. Exposures must be verified against [47] radiance/irradiance limits and classified under IEC 62471:2022 hazard groups, with session logs and calibration certificates retained to ensure compliance and auditability.

By consolidating international standards [46,47,48,49], these parameters are proposed as reproducible conditions for cone-targeted neuromodulation. They form the implementation backbone onto which our filter classification and condition-specific mapping are applied. Having established this proposed operational framework, the subsequent section presents the optical characterization of monochromatic and combined filters, detailing their transmittance profiles, cone selectivity, and integration into condition-specific protocols.

### 2.3. Filter Characterization

This section does not introduce new spectral measurements; instead, it consolidates and applies previously validated data from four published studies [1,2,9,10]. In those works, spectral transmittance and cone-response values were obtained for eleven monochromatic and twenty two combined filters. For the present protocol, these published cone-response percentages, including cross-talk to non-targeted cones, were systematically re-analyzed using Python 3.13.5-based routines (NumPy, SciPy) to screen filter performance from the verified spectral data.

Note. The filter designations used throughout this section (e.g., Omega, Mu, Alpha, Depressant) correspond to traditional clinical terminology widely recognized in visual-therapy practice. Each is cross-referenced with its measured spectral transmittance, dominant wavelength, and cone-excitation percentage (Table 3, Table 4, Table 5 and Table 6), ensuring standardized physical interpretation and full reproducibility. Filters were classified as efficient or non-efficient based on quantitative cone-excitation modeling derived from our previous spectrophotometric analyses. A filter was considered efficient when modeling demonstrated ≥5% relative excitation in at least one targeted cone class under standard photopic conditions, ensuring physiologically meaningful and selective activation. Filters showing <0.01% excitation for all cone classes were classified as inefficient because they produced negligible activation, whereas combined filters were retained only when they yielded ≥5% excitation in a targeted cone class. Combinations producing <1% excitation across all cones were discarded as clinically irrelevant. These specific cut-offs derive from established cone-excitation conventions in visual photometry and colorimetry [30,48]. A differential excitation of about 5% represents the minimal physiologically meaningful bias that exceeds instrumental variability (≈2–3%) while preserving cone selectivity. Thresholds above 10% would widen the effective spectral bandpass, increasing overlap among neighboring cone sensitivities (e.g., M/L) and reducing isolation. The 1% and 0.01% limits correspond respectively to negligible and null activation levels within modeling precision. Consistent with earlier findings, filter combinations exhibited reduced overall transmittance and diminished cone selectivity. Therefore, only six representative pairs were retained for inclusion in the clinical framework. Table 3, Table 4, Table 5 and Table 6 summarize the filters selected for targeted S-, M-, and L-cone stimulation. Each table is grounded in experimental characterization and provides a reproducible mapping of filters to cone classes, ensuring that the protocol builds directly on validated measurements and computational screening rather than heuristic choice. Filters producing minimal cone excitation (<10%) were retained only as reference examples, as they may hold exploratory relevance in patients with heightened neural sensitivity—such as those with brain injury or post-concussive conditions—where low-intensity spectral stimulation could be better tolerated. This remains a working hypothesis requiring empirical verification before clinical implementation.

#### 2.3.1. Monochromatic Filters

Monochromatic filters constitute the foundation of cone-targeted stimulation, as their narrowband transmission enables direct mapping of spectral properties to cone-selectivity indices. The present protocol draws on prior spectrophotometric characterization of eleven filters, in which transmittance spectra and modelled cone-excitation percentages were quantified and validated across multiple studies [1,2,9,10]. From this dataset, filters were systematically screened using computational routines to identify those offering the most effective balance of selectivity and transmission. Table 3, Table 4, Table 5 and Table 6 summarize the retained filters, detailing their spectral properties, cross-talk to non-target cones, and practical clinical applications.

##### S-Cone Filters

The spectral properties and cone-selectivity of S-cone–targeting filters are summarized in Table 3.

**Critical interpretation**

- **Omega.** Provides the most selective S-cone activation (13.48%) with negligible M (0.04%) and L (0.16%) cross-talk. Despite low transmittance (19%), it is the most reliable option for isolating short-wavelength responses in diagnostic protocols and baseline assessments. Clinical use: best suited for short blocks (8 min total at 250–350 lx, morning/early-day) where precise S isolation is required.
- **Lambda.** Achieves higher drive (40.10% S) while maintaining minimal cross-talk, M (1.25%) and L (0.22%). With 50.87% transmittance, it balances selectivity and power, making it suitable for sustained therapeutic protocols. Clinical use: appropriate for therapy sessions requiring moderate S stimulation, using block design from Table 2 (2 min × 4, with rests) to prevent adaptation.
- **Upsilon.** Offers selective S stimulation (35.68%) with very low spillover M (1.55%), L (0.03%) and moderate transmittance (44.70%). Clinical use: useful for training programs where luminance control is critical; can be combined with gradual ramp-up and diffuser use to reduce glare.
- **Neurasthenic.** Provides modest S activation (25.31%) with low M spill (0.22%) but substantial L contamination (8.16%). Clinical use: not suitable for strict isolation but may be employed in exploratory or low-intensity protocols where L involvement is tolerable. Should be applied at lower illuminance, with short blocks and diffusers to limit L-driven luminance effects.
- **Pi.** Strong S activation (66.10%) with moderate M spill (13.14%) and negligible L (0.05%). High transmittance (76.05%) allows robust stimulation. Clinical use: appropriate for advanced training phases seeking strong S input, provided block durations and rests are respected to prevent over-adaptation.
- **Depressant.** Maximizes S activation (72.54%) but introduces high M (23.01%) and L (7.35%) cross-talk. Clinical use: only suitable for short-term experimental protocols where maximum S drive is required despite contamination; must remain within illuminance and timing guardrails and be carefully monitored for visual fatigue.

Taken together, Omega is the reference filter for strict S-cone isolation, while Lambda and Upsilon offer the best balance between selectivity and power for therapeutic use. Pi and Depressant provide strong S activation but at the cost of specificity, limiting their role to advanced or short-term protocols. Neurasthenic, with notable L contamination, is best restricted to exploratory or low-intensity applications. This hierarchy aligns filter choice with the operational guardrails defined in Table 2, ensuring both reproducibility and clinical safety.

##### M-Cone Filters

The spectral properties and cone-selectivity of M-cone–targeting filters are summarized in Table 4.

**Critical interpretation**

- **Mu.** Provides the most selective M-cone stimulation (12.08%) with minimal S (0.49%) and L (0.01%) cross-talk. Its moderate transmittance (34.76%) limits absolute drive but ensures purity, making it the filter of choice for experimental paradigms requiring isolation and for clinical contexts where precise M-cone input is needed (e.g., vergence or visuomotor testing). Clinical use: apply under the M-cone operational window from Table 2 (400–500 lx, 16–20 min total, with rests) to maintain stability while avoiding L-driven confounds.
- **Stimulant.** Achieves very high M activation (60.83%) but at the cost of strong L contamination (34.06%) and minor S spillover (1.90%). With high transmittance (89.17%), it can drive robust responses but sacrifices specificity. Clinical use: suitable for dynamic training phases where strong magnocellular activation is desired and some parvocellular/L involvement is acceptable. Must be carefully timed and monitored to prevent over-stimulation.
- **Delta.** Delivers strong M excitation (51.26%) with significant L contamination (34.28%) and small S involvement (2.75%). High transmittance (87.99%) supports robust drive. Clinical use: appropriate in combined-filter protocols where luminance control is prioritized, but requires strict block timing and illuminance control to prevent L-cone dominance.
- **Theta.** Provides moderate-to-high M drive (47.12%) with comparable L contamination (34.30%) and negligible S spill (1.02%). Transmittance is high (86.57%). Clinical use: useful in intermediate therapy phases where pure isolation is not critical, but where sustained luminance input can support fixation and visuomotor stability.

**Comparative takeaway.** Mu is the only filter that offers true M-cone selectivity and should be prioritized in isolation paradigms. Stimulant, Delta, and Theta provide stronger M activation but are consistently compromised by substantial L cross-talk (34%), limiting their utility for precise experiments. Nonetheless, they may have value in clinical or rehabilitative programs where robust total cone engagement is acceptable, provided block design and illuminance guardrails from Table 2 are strictly observed.

##### L-Cone Filters

The spectral properties and cone-selectivity of L-cone–targeting filters are summarized in Table 5.

**Critical interpretation**

- **Alpha.** Provides the cleanest L-cone isolation (8.50%) with negligible S (0.22%) and M (0.01%) cross-talk. Its relatively low excitation amplitude limits robustness, but its high transmittance (81.80%) ensures good light throughput. Clinical use: best suited for diagnostic protocols or baseline measurements requiring selective L stimulation. Should be applied within the L-cone operational window (500–650 lx, 20 min total, with ramp-up) to maximize comfort and foveal response.
- **Stimulant.** Generates strong L activation (34.06%) but is heavily confounded by M spillover (60.83%) and minor S involvement (1.90%). High transmittance (89.17%) facilitates strong drive. Clinical use: may be considered in short, controlled sessions where enhanced long-wavelength responsivity is desired, but only under strict block and illuminance control due to lack of specificity.
- **Delta.** Produces L activation (34.28%) with significant M contamination (51.26%) and some S spill (2.75%). High transmittance (87.99%) supports efficient drive. Clinical use: suitable for brief, high-intensity protocols, where some cross-activation can be tolerated; requires monitoring to prevent luminance-driven adaptation effects.
- **Theta.** Yields comparable L activation (34.30%) with substantial M spill (47.12%) and minor S involvement (1.02%). With high transmittance (86.57%), it delivers strong output. Clinical use: appropriate for intermediate therapy phases where balanced stimulation across L and M pathways is acceptable, supporting acuity and fixation stability rather than strict isolation.

**Comparative takeaway.** Alpha is the only filter offering true L-cone selectivity and should be prioritized in isolation paradigms. Stimulant, Delta, and Theta provide stronger L drive (34%) but at the cost of substantial M contamination, restricting their role to short or intermediate training phases where cross-activation is acceptable. This clinical hierarchy ensures that L-cone filters are applied within the safety and reproducibility guardrails defined in Table 2.

#### 2.3.2. Combined Filters

Whereas monochromatic filters maximize cone drive but often introduce significant cross-talk, combined filters are designed to attenuate amplitude while improving relative selectivity. Their characterization builds on the same spectrophotometric dataset used for the monochromatic set, with cone-response percentages derived from validated transmittance spectra [1,2,9,10]. Filter pairs were systematically screened with Python-based routines to identify combinations that bias S-, M-, or L-cone pathways while minimizing unwanted activation of non-target cones.

From the 22 possible filter pairings initially analyzed, only six produced a modeled cone-response above 5%. The remaining combinations yielded responses below 1% for all cone classes and were therefore excluded as clinically irrelevant. This threshold ensured that only pairings with meaningful physiological drive were retained, avoiding the inclusion of combinations whose spectral overlap was too weak to provoke any significant changes to the visual system and cortex.

This approach yielded six representative pairings that are summarized in Table 6. Unlike the monochromatic filters, which emphasize maximum drive, these combinations offer gentler but more controlled cone biases. This makes them particularly relevant in contexts where over-stimulation is a risk (e.g., migraine, traumatic brain injury, amblyopia rehabilitation) or where gradual sensitization is desirable. Their clinical value lies not in raw intensity but in providing targeted spectral contexts that can be integrated into carefully phased protocols.

**Critical interpretation (with clinical applications)**

- **Upsilon–Neurasthenic.** Provides attenuated S activation (9.72%) with near-null M (0.01%) and L (0.02%) cross-talk. Its low drive makes it unsuitable for sustained protocols but valuable as a sensitization block or diagnostic probe, particularly in patients where over-stimulation is a risk. Clinical use: morning/early-day exposure, short blocks at lower illuminance, useful as a gentle S-bias introduction.
- **Omega–Pi.** Yields similar S selectivity (9.39%) with minimal spillover, M (0.02%) and L (0.01%). Compared to monochromatic S filters, it sacrifices amplitude for purity. Clinical use: an alternative for diagnostic S isolation when the lowest possible contamination is required but not intended for high-drive therapeutic sessions.
- **Mu–Delta.** Produces selective M activation (6.40%) with negligible S (0.07%) and L (0.01%) cross-talk. This represents one of the rare near-isolating M conditions available. Clinical use: appropriate for research or clinical contexts targeting visuomotor coordination, applied at the standard M operational window (400–500 lx, 16–20 min total), though the weaker drive limits rehabilitative potency.
- **Mu–Theta.** Delivers a comparable M bias (5.22%) with almost no S (0.03%) or L (0.01%) involvement. Functionally interchangeable with Mu–Delta. Clinical use: selected based on patient tolerance or sequence design; offers flexibility in designing M-centric blocks without cross-contamination.
- **Delta–Theta.** Drives both M (36.87%) and L (30.58%) strongly, with cross-talk too pronounced for selective use. Clinical use: reserved for short, controlled “push blocks” when broad mid-to-long wavelength activation is justified (e.g., late-phase protocols seeking robust total cone engagement). Must be applied with strict duration and safety checks to prevent over-activation.
- **Alpha–Delta.** Provides a moderate L bias (7.70%) with negligible S (0.00%) and M (0.01%) cross-talk. Unlike high-power red filters, it minimizes M recruitment, giving a more controlled long-wavelength drive. Clinical use: suitable when selective L emphasis is needed (e.g., fixation or acuity tasks), although reduced amplitude limits its use to moderate-intensity contexts.

**Comparative takeaway.** Combined filters trade amplitude for precision, offering gentler but more controlled cone biases. Upsilon–Neurasthenic and Omega–Pi provide clean but attenuated S drive; Mu–Delta and Mu–Theta deliver rare near-M isolation; Alpha–Delta offers controlled L bias; and Delta–Theta functions only as a broad “push block.” Their utility lies in contexts where gradual sensitization, reduced glare, or minimized cross-activation is clinically preferable, complementing the high-drive but less selective monochromatic set.

The daily sequence of cone-specific sessions was organized in accordance with human circadian and photoreceptive physiology. S-cone–weighted sessions were conducted in the morning to coincide with the peak circadian sensitivity of melanopsin and short-wavelength pathways, thereby enhancing alertness and phase alignment. Exposure to short-wavelength light during early hours is known to advance circadian phase and increase cortical arousal [73,74,75]. Conversely, L-cone–weighted sessions were delivered in the afternoon, when reduced melanopsin responsivity minimizes circadian disturbance while favoring visual–motor integration and sustained attention [75,76]. This schedule aligns each spectral weighting with the natural time-of-day modulation of human photoreception and alertness, ensuring physiological coherence across therapy stages.

Taken together, the characterization of monochromatic and combined filters defines the full optical toolkit available for cone-targeted stimulation. Monochromatic filters provide maximum drive but are constrained by cross-talk, while combined filters attenuate amplitude to achieve greater control and clinical tolerability. These complementary profiles ensure that stimulation parameters can be tailored both for precision diagnostics and for progressive therapeutic contexts. The following section translates these characterized filters into disorder-specific applications, outlining how each can be integrated into standardized clinical protocols.

### 2.4. Condition-Specific Neuromodulation Protocols

The characterized filters described in Section 2.3 provide the optical foundation for cone-targeted neuromodulation. Section 2.4 translates these spectral tools into disorder-specific clinical protocols, integrating cone physiology, adaptation dynamics, and the safety guardrails defined in Section 2.2. These protocols are presented as mechanistic constructs rather than efficacy demonstrations, designed to illustrate how cone-specific stimulation can be aligned with the pathophysiological features documented in each condition. Protocol design was guided by established neurophysiological and clinical evidence, but validation to date remains limited to conventional approaches such as binocular vision therapy and medical intervention. Accordingly, these constructs should be regarded as physiologically grounded frameworks that outline translational potential, pending targeted clinical evaluation. Each protocol specifies the filter set, operational parameters, and session structure most appropriate for targeting the visual and cortical mechanisms implicated in a given condition, thereby providing standardized, evidence-based blueprints for systematic testing in future clinical trials.

#### 2.4.1. Amblyopia

Core deficits in amblyopia include reduced visual acuity, impaired stereopsis, and abnormal binocular balance with suppression, as documented in clinical reviews and psychophysical studies [77,78]. Binocular and dichoptic training strategies that reduce suppression and promote functional recovery have demonstrated translational and clinical benefits [79,80,81]. From a physiological perspective, parvocellular L/M-opponent pathways underpin high-acuity foveal processing [14,65], mid-wavelength inputs contribute to dorsal-stream visuomotor functions, and short-wavelength pathways engage ipRGC and koniocellular routes that influence arousal and non-visual responses [7,82]. These links are highlighted here to align cone-targeted stimulation with the mechanisms most relevant to amblyopia, while remaining framed strictly within physiological rationale, not presented as therapeutic evidence.

#### 2.4.2. Esotropia

The pathophysiology of esotropia is characterized by excessive convergence due to an abnormally high accommodative–vergence (AC/A) ratio, reflecting abnormal accommodation–convergence coupling, with central control mediated by brainstem and midbrain vergence circuits [83,84,85]. Neuroimaging evidence further shows abnormal spontaneous cortical activity in strabismus and amblyopia, reinforcing the central basis of these dysfunctions [86]. Short-wavelength stimulation modulates parasympathetic output via ipRGC projections to the olivary pretectal nucleus and Edinger–Westphal complex, and S-cone signals contribute to the pupillary light reflex; these mechanisms provide a physiological basis for considering short-wavelength bias as a potential lever, without implying clinical efficacy [39,82]. In contrast, long-wavelength input increases accommodative drive [87], supporting the rationale for minimizing L-cone bias in esotropic protocols. Mid-wavelength M-cone stimulation is linked to dorsal-stream visuomotor functions [33,88], which may contribute to calibration of vergence responses. Together, these mechanistic associations justify a cone-targeted framework for esotropia, while remaining within the scope of physiological rationale rather than clinical validation.

#### 2.4.3. Exotropia

Current management of exotropia typically involves observation, orthoptic exercises, prisms, or surgery, but evidence syntheses indicate that these approaches have limited long-term efficacy, particularly for intermittent forms [89,90]. Suppression and abnormal binocular integration are consistently documented in psychophysical studies, highlighting altered sensory correspondence as a defining feature [91]. From a mechanistic perspective, cone-opponent pathways provide a framework for considering targeted stimulation. L/M parvocellular inputs contribute to fine spatial detail and binocular alignment [14,65], while dorsal-stream integration of mid-wavelength signals supports visuomotor stability and dynamic fusion [33,88]. Short-wavelength S-cone signals, by modulating arousal and non-visual responses, may indirectly influence binocular control under specific conditions, though these links remain theoretical. These associations are not presented as therapeutic claims but as physiological rationale: by mapping cone-selective stimulation to the sensory and motor features of exotropia, filter-based neuromodulation protocols can be formulated as translational constructs for future testing.

#### 2.4.4. Hypertropia

Hypertropia reflects vertical ocular misalignment with etiologies linked to dysfunction of midbrain and brainstem gaze circuits, including the rostral interstitial nucleus of the medial longitudinal fasciculus (riMLF), interstitial nucleus of Cajal (INC), superior colliculus (SC), and cerebellar inputs [85,92,93]. The SC has long been recognized as a hub for orienting responses, with classical physiology showing that short-wavelength inputs can influence its activity [94]. In parallel, M-cone–biased signals contribute to dorsal-stream visuomotor timing and spatial calibration [33,88]. These associations are highlighted here not as therapeutic claims, but as mechanistic grounds for emphasizing M- over S-cone stimulation in the design of translational protocols for hypertropia.

#### 2.4.5. Hypotropia

Hypotropia involves vertical ocular misalignment associated with dysfunction in the premotor network for vertical alignment, which includes midbrain centers, the superior colliculus, and cerebellar contributions [85,92,93]. Short-wavelength input engages ipRGC and koniocellular pathways projecting to the superior colliculus and pulvinar, with cortical influence documented in temporal-dynamic studies of S-cone signals [7]. In parallel, parvocellular L/M-opponent circuits underpin foveal acuity and fine spatial processing [14,65]. Together, these findings provide a mechanistic rationale for considering short S-biased exposures alongside L/M-targeted inputs in protocols for hypotropia, while remaining strictly within the scope of physiological association rather than clinical validation.

#### 2.4.6. Attention-Deficit/Hyperactivity Disorder (ADHD)

ADHD is associated with large-scale network alterations and oculomotor dysfunction, including fixation instability and impaired saccadic control, as consistently reported in neuroimaging and behavioral studies [18,95,96,97]. Electrophysiological studies repeatedly document atypical alpha–theta dynamics across children and adults with ADHD, highlighting disrupted cortical oscillatory balance [98].

Beyond cognitive and oculomotor domains, circadian misalignment is a recurrent physiological feature of ADHD, characterized by delayed dim-light melatonin onset, evening chronotype, and irregular sleep–wake rhythms [99,100]. These disturbances have motivated exploration of chronobiological and light-based interventions—such as bright-light therapy and spectral-modulation paradigms—designed to advance circadian phase and stabilize arousal regulation [101,102].

From a mechanistic perspective, intrinsically photosensitive retinal ganglion cells (ipRGCs) and S-cone–driven pathways project to thalamo-cortical and limbic structures implicated in arousal, attention, and temporal synchronization [76,103,104]. Spectral-integration studies demonstrate that melanopsin- and L-cone–induced pupil constriction is modulated by S- and M-cones [105] and that melanopsin activation enhances image persistence and cortical alertness [106]. Recent reviews confirm that ipRGCs exert diverse non-image-forming functions linking light input to cognitive and emotional regulation [107]. Together, these observations connect visual, oculomotor, and circadian mechanisms, providing the neurofunctional rationale for the S/M/L-cone triad outlined in Table 7. This represents a unified, safety-compliant model for cone-specific light stimulation and adaptive neuromodulation in ADHD. The framework offers a physiologically grounded hypothesis linking cone-specific modulation to attentional and arousal networks and should be regarded as a conceptual template for future empirical validation, rather than a demonstrated therapeutic approach. In current visual-neuromodulation and therapy protocols, multiple monochromatic or combined filters are often applied simultaneously, making it unclear which spectral ranges effectively modulate cortical activity. Our previous spectrophotometric studies, conducted in collaboration with the National Center of Metrology, demonstrated that several commercially used filters produced relative cone-excitation values below 0.01%, indicating negligible photoreceptor activation. This systematic analysis enabled the identification and retention of only those filters with verified cone-specific excitation and reproducible neurophysiological responses. Building on this evidence, the present framework reconstructs a data-driven protocol using these validated filters and, for the first time, defines the key optical parameters—illuminance, distance, and exposure geometry—that were absent in earlier therapeutic devices. This structure bridges optical precision with cortical modulation, ensuring both reproducibility and physiological plausibility.

#### 2.4.7. Comparative Mechanistic Mapping

This synthesis translates physiological associations into methodological blueprints, clarifying why cone weighting differs across conditions and how these rationales inform protocol design.

**Interpretation of the mechanistic mapping.** Table 7 integrates the cone-related mechanisms outlined in Section 2.4.1, Section 2.4.2, Section 2.4.3, Section 2.4.4, Section 2.4.5 and Section 2.4.6 across six clinical conditions. It highlights how S-, M-, and L-cone pathways contribute to distinct domains of visual processing, including foveal fixation, visuomotor integration, vertical gaze control, and circadian–arousal regulation. This mapping represents a methodological synthesis grounded in physiological rationale, offering a reproducible framework for future clinical evaluation.

Across conditions, L-cone input is consistently associated with parvocellular-driven foveal fixation and acuity, M-cone input with visuomotor and vergence stability through dorsal-stream circuits, and S-cone input with arousal and circadian modulation via ipRGC-linked pathways. These complementary functions explain the cone-weighting patterns observed in amblyopia, strabismus subtypes, and ADHD, while clarifying why different conditions emphasize different cones (e.g., minimizing L bias in esotropia to avoid accommodative drive, or combining S+M+L in ADHD to address multisystem deficits).

This synthesis provides a structured methodological framework for designing condition-specific protocols grounded in visual physiology. Its purpose is to define translational constructs for systematic clinical testing, while recognizing that validation of cone prioritization across disorders will require prospective, controlled trials before therapeutic implementation can be recommended.

Building on this foundation, Section 2.5 addresses treatment duration. The proposed time frames are not outcomes of the present study but are derived from two complementary sources: (i) our previously published interventions with monochromatic and combined filters, which consistently employed 20-session protocols [1,2], and (ii) independent clinical and translational evidence, including randomized controlled trials, regulatory submissions, and systematic reviews in amblyopia, strabismus, and ADHD [77,89,101,108]. Together, these studies provide validated temporal benchmarks that anchor the proposed light-based framework within established therapeutic standards, ensuring methodological reproducibility and scientific rigor, while acknowledging that further clinical validation remains necessary.

### 2.5. Duration Framework for Cone-Targeted Neuromodulation

Beyond spectral and safety parameters, an essential methodological question is the overall time frame required for light-based interventions. The framework presented here integrates two complementary sources of evidence: (i) our own published interventional studies in strabismus and amblyopia, which consistently employed 20-session protocols with monochromatic and combined filters [1,2], aligning with the historical syntonic precedent of approximately 20 sessions; and (ii) external randomized and systematic evidence from amblyopia, strabismus, and ADHD, which provides condition-specific benchmarks for therapy duration.

Amblyopia: Our neuromodulation studies established a 20-session framework, demonstrating reproducibility of cortical and functional responses [1,2]. Randomized trials of binocular and digital therapies typically extend treatment to 12–16 weeks, with benefit sustained for up to one year [80,81,108]. Classic patching and pharmacological penalization require longer exposures, often several months [77]. Thus, 20 sessions provide a methodological baseline, expandable to 12–16 weeks when functional or electrophysiological improvement is confirmed.

Strabismus: Non-surgical interventions remain heterogeneous. Cochrane reviews of intermittent exotropia highlight the absence of a standardized treatment length, though orthoptic and binocular programs frequently span 8–12 weeks [89,90]. Within this context, the 20-session baseline derived from our protocols can be expanded into longer courses of 8–12 weeks when improvements in vergence ranges, suppression, or fixation stability are documented. Other forms (esotropia, hypertropia, hypotropia) lack direct duration data, and any proposed schedules must be regarded as investigational.

ADHD: Published trials report effective courses as short as 2–3 weeks [101], with pilot randomized controlled trial (RCTs) and preventive studies extending to 10 weeks when circadian or attentional outcomes warrant [109,110,111]. For cone-targeted neuromodulation, this suggests short-duration regimens of 2–3 weeks, with optional extension to 10 weeks based on objective endpoints.

**ynthesis.** Taken together, these data support a convergent framework:

- A 20-session core for amblyopia and strabismus, expandable to 12–16 weeks for amblyopia and 8–12 weeks for strabismus.
- A shorter 2–3-week core for ADHD, with extension up to 10 weeks when justified.

This condition-specific model situates cone-targeted light therapy within the broader therapeutic literature, providing reproducible methodological guidance that is supported by converging neurophysiological and clinical evidence, while stopping short of implying proven efficacy. Table 8 summarizes these benchmarks, offering trial designers an evidence-based reference for treatment duration across the three principal conditions studied.

In summary, this section fuses cone-specific physiology with validated duration evidence to establish structured temporal parameters, ensuring reproducibility and methodological rigor in the proposed neuromodulation protocols.

## 3. Results

The present framework consolidates filter selectivity, calibrated light-delivery parameters, and condition-specific therapeutic priorities into an integrated clinical proposal for cone-specific neuromodulation. The emphasis is explicitly translational: this proposal establishes how the optical and dose-defined values detailed in the Section 2 provide the scientific foundation required to operationalize reproducible, condition-specific protocols for amblyopia, strabismus, and ADHD—ensuring that the framework can and should be adopted in future research and clinical applications.

### 3.1. Filter Selectivity

Spectrophotometric data identified three monochromatic filters that are proposed as the most reliable for single-cone bias: Omega for S-cones (440 ± 15 nm), Mu for M-cones (530 ± 15 nm), and Alpha for L-cones (570 ± 15 nm). High-drive filters (Pi, Depressant, Stimulant, Delta, Theta) provide stronger stimulation but also introduce cross-activation; their use is therefore proposed to be limited to brief, high-intensity blocks applied under strict methodological control. Combined filters are proposed where more nuanced biasing is required: S-bias (Upsilon–Neurasthenic, Omega–Pi), M-bias (Mu–Delta, Mu–Theta), L-bias (Alpha–Delta), and broad M+L (Delta–Theta). Delta–Theta demonstrates minimal selectivity and should not be used for cone isolation, but only as a brief augmentative block with explicit methodological justification. These proposals align directly with the spectral transmission profiles and methodological safeguards outlined in the Section 2.

### 3.2. Light Parameters and Time Framework

Stimulation parameters are proposed to align with the validated operational windows defined in Section 2 (Table 2) and are organized into condition-specific frameworks in Table 9. Within this proposal, S-cone stimulation is scheduled in the morning at lower illuminance with shorter blocks, M-cone stimulation at moderate daytime levels to support visuomotor stability, and L-cone stimulation at higher daytime illuminance with longer blocks to reinforce fixation. All proposed exposures assume spectroradiometer-verified narrowband LEDs, corneal-plane dose verification, and diffuser safeguards, with uniform safety rules (DC or high-frequency PWM ≥ 1 kHz, exclusion of 15–25 Hz flicker, pre-screening for photosensitivity, and age-adjusted working distances). Treatment courses are framed as standardized baselines—20 sessions for amblyopia and strabismus (extendable to 12–16 or 8–12 weeks, respectively) and 2–3 weeks for ADHD (extendable up to 10 weeks). This framework situates session-level parameters within reproducible, clinically relevant schedules that now require prospective validation.

### 3.3. Integrated Framework

The standardized spectral, temporal, and safety parameters established above provided the structural basis for translating cone-specific selectivity into applied clinical protocols. These elements were consolidated into an integrated framework, summarized in Table 9 and detailed in condition-specific extensions (Table 10, Table 11, Table 12 and Table 13).

Table 9 compiles all validated filter options, including combined pairings, to provide a comprehensive reference. In contrast, the condition-specific (Table 10, Table 11, Table 12 and Table 13) are proposed to prioritize only those filters with a mechanistic rationale for each disorder; combined filters are included selectively where their balance of drive and selectivity is relevant (e.g., ADHD).

The integrated framework is proposed to align cone-specific pathways with the functional deficits characteristic of each condition. For amblyopia, L-cone stimulation is proposed to reinforce central fixation and acuity, supported by M-cones to stabilize visuomotor integration, with S-cones as brief adjuncts. For esotropia, S-cone bias is proposed to reduce accommodative load, M-cones to enhance vergence stability, and L-cone stimulation should generally be avoided to prevent accommodative overdrive. For exotropia, L-cone stimulation is proposed to stabilize fixation, M-cones to support motor integration, and S-cone input should be restricted to brief morning exposures to avoid destabilizing fusion. For ADHD, sequential stimulation of all three cones (S→M→L) is proposed: S-cones for arousal and circadian alignment, M-cones for visuomotor tracking, and L-cones for sustained fixation and attentional control.

Clinically, this framework is intended to provide a reproducible, safety-based rationale for tailoring cone stimulation to the neurophysiological demands of each disorder as presented in Table 10, Table 11, Table 12 and Table 13.

Amblyopia protocols are proposed to emphasize L-cone–driven parvocellular input to reinforce central fixation and foveal acuity, with M-cone stimulation supporting visuomotor integration and binocular coordination. Brief, optional S-cone exposures may be applied to enhance cortical responsiveness but should remain deliberately constrained to avoid overstimulation. This balance is intended to address the core deficits of amblyopia—impaired acuity, stereopsis, and suppression—by strengthening central visual pathways while facilitating fusion. Protocols are designed on a 20-session baseline, with extension to 12–16 weeks when functional or electrophysiological gains are anticipated.

Within this framework, the proposed filter set specifies:

- **Alpha (L-cone):** Primary filter to reinforce central fixation and acuity.
- **Mu (M-cone):** Supportive filter for visuomotor integration.
- **Omega (S-cone):** Optional, brief adjunct to enhance cortical responsiveness.
- **Clinical logic:** Use Alpha + Mu as the mainstay; add Omega sparingly in short morning blocks to avoid overstimulation.

Esotropia protocols are proposed to prioritize S-cone stimulation, which may lower accommodative load via parasympathetic modulation and thereby alleviate convergence stress. Secondary M-cone activation is proposed to reinforce vergence stability and visuomotor timing. L-cone input is contraindicated, as long-wavelength stimulation is likely to heighten accommodative drive and risk worsening esodeviation. Clinically, this selective emphasis is intended to mitigate accommodative overdrive and promote binocular stability without reinforcing convergence mechanisms. Protocols are designed on a 20-session baseline, with extension to 8–12 weeks when improvements in vergence, suppression, or fixation are anticipated.

Grounded in this rationale, the proposed filter set specifies:

- **Omega (S-cone):** Primary filter to lower accommodative load.
- **Mu (M-cone):** Secondary filter for vergence stability.
- **Contraindicated:** Alpha (L-cone), due to risk of accommodative overdrive.
- **Clinical logic:** S-first approach with optional Mu support; avoid any long-wavelength bias.

Exotropia protocols are proposed to emphasize L-cone stimulation to stabilize foveal fixation and enhance central acuity, thereby reducing suppression and abnormal binocular integration. M-cone activation is proposed to complement this by reinforcing vergence stability and visuomotor control. S-cone exposure should be limited to brief, morning-only blocks that may boost arousal but risk destabilizing fusion if prolonged. Clinically, this strategy is intended to strengthen fixation and fusional stability by directly targeting the physiological mechanisms most affected in exotropia. Protocols are designed on a 20-session baseline, with extension up to 8–12 weeks when measurable gains in alignment or suppression control are anticipated.

In line with these mechanisms, the proposed filter set specifies:

- **Alpha (L-cone):** Primary filter to stabilize fixation and promote convergence.
- **Mu (M-cone):** Secondary filter to support visuomotor control.
- **Omega (S-cone):** Restricted to brief morning exposures only.
- **Clinical logic:** Rely on Alpha + Mu for core treatment, with Omega limited to short blocks to prevent fusion disruption.

**Other strabismic entities.** Hypertropia and hypotropia, are not included in the present framework because no validated evidence currently defines their cone priorities, light parameters, or treatment duration. While vertical strabismus may engage distinct fusional and torsional mechanisms, any cone-targeted neuromodulation protocols for these entities remain investigational. Their incorporation should await dedicated optical and clinical studies able to establish selective filter use, safety windows, and reproducible temporal frameworks. Until such data exist, proposed parameters for vertical deviations should be considered exploratory and restricted to research protocols.

ADHD protocols are proposed to implement a sequential S→M→L approach, aligned with the functional domains most impaired in the disorder. Morning S-cone stimulation is proposed to enhance arousal and circadian alignment, M-cone input to support visuomotor control and oculomotor tracking, and L-cone activation to reinforce sustained foveal fixation for attentional stability. This sequence is intended to target the triad of ADHD deficits—arousal regulation, visuomotor performance, and sustained attention—within a reproducible, safety-controlled framework. Protocols are designed on a 2–3 week baseline, with extension up to 10 weeks when objective improvements are anticipated.

Combined filters (e.g., Mu–Delta, Alpha–Delta) are proposed for selective use to balance drive and selectivity. While monochromatic filters maximize absolute cone activation, they also increase cross-talk. Combined filters reduce overall amplitude but provide more controlled, cone-biased stimulation. This trade-off is particularly relevant in ADHD, where maintaining balanced selectivity across S, M, and L pathways is more critical than maximizing single-cone drive. Their use is therefore methodological, intended to preserve reproducibility and tolerability, rather than to suggest superior clinical efficacy.

The ADHD protocol is proposed to require longer total exposure (44 min) because all three cone pathways (S, M, L) are engaged sequentially, each contributing distinct physiological functions. In contrast, strabismus protocols emphasize only one or two cone classes. Thus, the longer duration reflects broader mechanistic coverage, not increased dosage per cone.

Building on this rationale, the proposed filter set specifies:

- **Sequential Omega → Mu → Alpha** for S-, M-, and L-cone activation, respectively.
- **Combined options** (e.g., Upsilon–Neurasthenic, Mu–Delta, Alpha–Delta) only when moderated drive is needed for tolerability.
- **Clinical logic:** Sequential pathway stimulation (≈44 min total) balances arousal, visuomotor integration, and sustained attention. Combined filters may reduce cross-talk but are not superior to monochromatics.

## 4. Discussion

To our knowledge, this is the first comprehensive clinical protocol for cone-specific neuromodulation, built on a coherent body of evidence from our previous experimental and clinical studies. Unlike earlier empirical approaches to light stimulation [112], which lacked spectral verification or outcome measures, our framework integrates detailed filter classification, neurophysiological validation, and condition-specific logic into a reproducible roadmap. By combining our data with complementary research on S-, M-, and L-cone pathways, we provide the first scientifically justified foundation for cone-based visual therapy.

Within the wider field of neuromodulation, this protocol reflects the shift toward interventions that are parameterized and mechanism-based. Noninvasive stimulation research has shown that carefully defined parameters can target neural circuits with measurable physiological and behavioral effects [113]. Our cone-specific design applies this principle by adapting established standards—spectral peaks, illuminance ranges, and block timing mapped to retinal and subcortical pathways—together with safety rules drawn from international guidelines: DC or high-frequency PWM ≥ 1 kHz, avoidance of 15–25 Hz, optional 32–40 Hz modulation only in research contexts, diffuser use, and age-adjusted working distances. These adaptations promote reproducibility and patient comfort across clinical profiles and age groups.

From a sensory-retinal perspective, converging human and rodent studies demonstrate that photoreceptor-specific drives produce distinct cortical, pupillary, and behavioral responses. Melanopsin contrast enhances image persistence in humans [106], while silent-substitution paradigms reveal opponent/complementary influences of cones and melanopsin on pupil dynamics [105]. Circuit-level work identifies ipRGC projections that mediate acute light-induced sleep regulation [114], and recent reviews emphasize their broad non–image-forming functions [107]. Collectively, these findings justify treating the S-, M-, and L-cone channels—and their interactions with melanopsin—as distinct physiological levers informing clinical dosing. Our classification operationalizes this principle: Omega, Lambda, Neurasthenic, and Upsilon for S-cones; Mu for M-cones; Alpha for L-cones; and Stimulant, Pi, Delta, Theta, and Depressant as overlapping/transitional filters, with six combined options (e.g., Omega–Pi, Mu–Delta, Alpha–Delta) to enable balanced or graded stimulation [10].

Although therapeutic efficacy remains to be demonstrated in controlled trials, the protocol consolidates reproducible parameters already supported by converging neurophysiological and clinical findings, including our previous studies. This provides a strong rationale for its acceptance as a structured foundation for future research. Cone-selective values and dose ranges were derived from controlled optical characterizations and validated through prior studies, then consolidated into Table 9 as a reproducible roadmap rather than a prescriptive guideline. The framework remains condition-specific and consistent with our findings: L- and M-cone emphasis in amblyopia (fixation and visuomotor integration), S-cone priority with L-avoidance in esotropia (reduction of accommodative load), L-cone stabilization with M support in exotropia (fixation and fusion), and an integrated S→M→L sequence in ADHD (arousal, visuomotor, sustained fixation). Evidence for vertical strabismus remains sparse, and any cone-targeted regimen should be regarded as investigational.

An important dimension of standardization is the treatment timeframe. We propose a 20-session baseline for amblyopia and strabismus—extendable to 12–16 weeks in amblyopia and 8–12 weeks in strabismus—together with a shorter 2–3 week baseline for ADHD, extendable up to 10 weeks. This contrasts with fragmented durations reported in the literature: amblyopia patching or penalization often requires several months [77], whereas digital and binocular therapies achieve benefits with 12–16 week courses [81,115,116,117,118]. For strabismus, Cochrane reviews highlight the absence of standardized schedules, with vision therapy programs spanning 8–12 weeks [89,119]. In ADHD, bright-light protocols show efficacy in as little as 2–3 weeks [101], but can extend to 10 weeks for circadian and attentional outcomes [109,110,111,120]. By distilling these heterogeneous precedents into condition-specific schemes, our proposal introduces a methodological anchor that is shorter and more reproducible than traditional regimens.

Historically, light-based interventions in both visual and neurological conditions were applied in broad, non-standardized ways—such as colored overlays, phototherapy lamps, or filter-based approaches—largely empirical in nature and adopted because they were recommended, not because their mechanisms were understood [112]. Although some studies suggested that filters could induce changes in the visual system and even cortical responses, the underlying neurological rationale remained unknown, and the spectral properties of the filters themselves were not characterized until recently. These approaches often produced inconsistent outcomes. By contrast, the present framework introduces optical verification, cone-specific targeting, and explicit safeguards, transforming empirical practice into an auditable system. Operationally, it is also more cost-effective: our previous analysis of 33 filters showed that most delivered negligible cone stimulation (<0.01%) [10]. By reducing the toolkit to eleven monochromatic and six combined filters, each with reproducible cone-selective impact, the protocol simplifies therapy while enhancing reproducibility. Combined filters are optional, used primarily for adaptation or multimodal sequences, since their cone-driving power is lower, but they preserve tolerability.

Preliminary clinical evidence underscores the need for this standardization. A randomized trial reported greater improvements in visual acuity with broad-spectrum light compared to occlusion [11]. Other studies showed that patching combined with therapy is more effective than patching alone [121], while systematic reviews confirm that non-specific phototherapy remains low-certainty and empirical [121]. Together, these findings highlight the limitations of older methods and the need for cone-specific, filter-verified protocols such as ours.

Although further verification in clinical trials is required, this work introduces a clinically logical framework grounded in existing evidence and reproducible methodology. Its value lies in offering a scientifically anchored basis that now requires prospective appraisal. It provides, to our knowledge, the first structured and reproducible basis for applying light stimulation in amblyopia, strabismus, and ADHD. Future trials must evaluate its impact in patient populations and combine functional outcomes with neurophysiological markers such as qEEG, VEPs, and fMRI. Only then can the therapeutic potential of this filter-based approach be fully determined. Although the protocol specifies diffuser use, corneal-plane dose verification, and α-opic EDI calculations, these procedures are clinically replicable. Daily verification can be performed with a calibrated lux meter and fixed diffuser, while periodic spectroradiometric checks establish device-specific conversion factors that allow routine use of photometric measurements in standard clinical settings. Thus, the safeguards and dosing rules proposed here do not require laboratory infrastructure but can be implemented with readily available clinical instruments.

Relative to external comparators, repeated low-level red light (RLRL) has demonstrated efficacy in pediatric myopia [122,123], though cone-density changes have also been reported [124]. These results highlight the need for dose ceilings and structured follow-up. Our framework addresses such concerns by defining illuminance windows, rest intervals, and safety safeguards. Other work explored blue or green light for circadian and cognitive modulation, including prefrontal activation during working memory tasks [125], but lacked cone-specific dosing and spectral verification. This further distinguishes our approach.

### Strengths, Limitations and Future Directions

In agreement with our previous studies, this framework does not propose light stimulation as a standalone therapy but as a complementary module within active visual training, where controlled photic modulation may enhance cortical coherence and facilitate neuroplastic adaptation. Nevertheless, the framework has both strengths and limitations. Although it does not present new clinical outcome data, it consolidates prior electrophysiological and optical findings into a unified protocol that requires confirmation through prospective, controlled trials with predefined endpoints. The present work does not include psychophysical or behavioral outcomes, as it was conceived as a pre-clinical methodological model to establish optical and neurophysiological reproducibility before functional effects are evaluated in controlled or randomized studies. Filter selection remains limited to the set of commercially available filters characterized in our previous research. While exclusion thresholds were applied to remove options with negligible modeled cone excitation, additional filters or custom spectra could further refine cone selectivity and should be systematically explored. Implementation depends on rigorous spectroradiometric verification, α-opic reporting, and compliance with photobiological and flicker-safety standards; centers lacking the appropriate measurement tools may not be able to reproduce the protocol precisely. The disorder-specific mappings, including those proposed for strabismus subtypes and vertical deviations, are mechanistic constructs grounded in known cone pathways and oculomotor physiology, and must be interpreted as hypotheses until tested in adequately powered clinical trials. Future studies should extend this framework to vertical strabismus—where the role of S- and M-cone pathways in vertical gaze circuits remains largely untested—and to neurodevelopmental conditions such as ASD, where chromatic sensitivity and sensory-integration profiles may provide valuable translational targets for cone-specific neuromodulation. The ADHD component of this framework is explicitly exploratory. Current evidence on light-based interventions in ADHD primarily involves chronobiological bright-light therapy and emerging neuromodulation strategies, not cone-selective filter approaches [109,111,126,127]. Accordingly, the proposed S–M–L sequence should be viewed as a conceptual template to guide future studies rather than a validated clinical recommendation.

Finally, inter-individual variability in photoreceptor sensitivity, lens and macular pigment density, and comorbid neurological or migraine conditions may influence response and warrants systematic evaluation in future work. In summary, this framework provides a reproducible and safety-compliant foundation for future clinical implementation, serving as a methodological reference for subsequent validation studies rather than a demonstration of therapeutic efficacy.

## Figures and Tables

**Table 1:** Evidence from four previously published experimental and clinical studies forming the empirical foundation of the proposed cone-specific neuromodulation framework. These peer-reviewed investigations quantified optical transmittance, cone-excitation percentages, and neurophysiological modulation (qEEG/VEP), defining the reproducible parameters that underpin the current methodological proposal.

Study	Participants	Modality	Key Findings	Evidential Significance
[1]	17 strabismus/amblyopia patients, 17 controls	qEEG during monochromatic filter stimulation	Pathological baseline oscillations (↑theta, ↓alpha, interhemispheric desynchrony) were acutely normalized during S-, M-, or L-biased stimulation (↑occipital alpha, restored interhemispheric synchrony)	Demonstrated that cone-targeted light can immediately reorganize pathological cortical activity, establishing causal capacity of wavelength-specific input to shift oscillatory dynamics
[2]	17 patients, 11 controls	qEEG + clinical outcomes after 20 stimulation sessions	Sustained ↑alpha power, improved VA, stereopsis, binocular alignment, and expanded VF; controls showed mild deterioration in stereopsis/phoria	Provided longitudinal evidence that repeated cone-specific stimulation drives durable cortical reorganization with parallel improvements in visual function, confirming therapeutic feasibility
[9]	12 participants (10 normal, 2 esotropia)	Pattern-reversal VEP with six monochromatic filters	Filter-dependent ↑N75/P100 latency and ↓amplitude; strongest modulation with Neurasthenic, Omega, and Mu	Confirmed wavelength-dependent modulation of early visual cortical responses, validating electrophysiological sensitivity of the visual system to monochromatic bias
[10]	Optical characterization (no patients)	UV–VIS–NIR spectrophotometry of 11 filters and 22 combinations	Quantified spectral transmittance, cone excitation percentages, and cross-talk for each filter configuration	Established the metrological standards required for reproducible and safe cone-specific stimulation, providing the technical foundation for proto

qEEG = quantitative electroencephalography; VA = visual acuity; VF = visual field.

**Table 2:** Protocol parameters for selective cone stimulation, including spectral bands, corneal illuminance, block design, session timing, temporal modulation safeguards, and α-opic reporting per CIE S 026.

Parameter	S-Cone (Short-λ)	M-Cone (λ)	L-Cone (Long-λ)
Spectral band/source	Narrowband LED 440 ± 15 nm (deep-blue; diffuser)	Narrowband LED 530 ± 15 nm (green; diffuser)	Narrowband LED 570 ± 15 nm (amber-red; diffuser)
Target corneal illuminance	250–350 lx	400–500 lx	500–650 lx
α-opic dose (CIE S 026)	S-opic EDI reported; melanopic EDI also reported	M-opic EDI reported; melanopic EDI also reported	L-opic EDI reported; melanopic EDI also reported
Block duration × repeats	2 min × 4	4 min × 4	5 min × 4
Rest between blocks	30–60 s	45–60 s	≈60 s
Total exposure time	8 min	16 min	≈20 min
Temporal modulation	DC or PWM ≥ 1 kHz; avoid 15–25 Hz; 32–40 Hz permitted only in controlled research (not clinical use).	Same	Same; gradual ramp-up first minute
Working distance & dose verification	Adjust geometry to meet targets; verify with lux meter + spectroradiometer; report distance/field/diffuser: children 40–50 cm/adults 50–60 cm/elderly 30–40 cm	Same	Same
Age adaptation	Children: lower lx (larger pupils). Older adults: slightly higher lx (lens absorption + smaller pupils): shorter blocks for <8 years old	Same	Same
Session timing	Morning/early day	Daytime/afternoon	Daytime; ramp-up

LED = light-emitting diode; lx = photopic illuminance at corneal plane; α-opic EDI = Equivalent Daylight Illuminance; PWM = pulse-width modulation; DC = direct current. Illuminance windows are anchored to ISO/CIE 8995-1:2025 and EN 12464-1:2021 (≈500 lx task areas; ≈300 lx circulation); gradual ramp = progressive increase in light intensity for comfort.

**Table 3:** Spectral properties and cone-selectivity of S-cone–targeting filters.

Filter	Cone	Role	Rationale	Cross-Talk	T (%)	Clinical Note
Omega	S	Maximum selectivity	Isolates S (13.48%) with almost no M/L	M ≈ 0.04%, L ≈ 0.16%	19.00	Best for pure S-isolation protocols or diagnostic purposes.
Lambda	S	Selectivity + moderate powery	Good S stimulation (40.10%) with very low spillover	M ≈ 1.25%, L ≈ 0.22%	50.87	Recommended for sustained S stimulation in therapy.
Upsilon	S	Alternative clean	S = 35.68% with excellent purity	M ≈ 1.55%, L ≈ 0.03%	44.70	Useful in training programs requiring fine luminance control.
Neurasthenic	S	Moderate selectivity; low M spill; notable L spill	S = 25.31% with minimal M contamination but elevated L 8.16%	M ≈ 0.22%, L ≈ 8.16%	36.02	Not recommended for strict isolation. Best applied at lower illuminance.
Pi	S	High drive, moderate selectivity	Strong S (66.10%) with tolerable M	M ≈ 13.14%, L ≈ 0.05%	76.05	Suitable for advanced phases seeking robust S activation.
Depressant	S	Maximum drive, low selectivity	Highest S (72.54%) but with significant M contamination	M ≈ 23.01%, L ≈ 7.35%	79.57	Only for short protocols where maximum S drive is needed.

Rationale percentages indicate modelled relative cone excitation, while transmittance (%), measured optical transmittance. Cross-talk entries indicate non-target cone activation. T (%) = Spectral transmittance at peak wavelength. S-cone = short-wavelength cone; M-cone = medium-wavelength cone; L-cone = long-wavelength cone.

**Table 4:** Spectral properties and cone-selectivity of M-cone–targeting filters.

Filter	Cone	Role	Rationale	Cross-Talk	T (%)	Clinical Note
Mu	M	Maximum selectivity	M drive (12.08%) with S and L almost null	S ≈ 0.49%, L ≈ 0.01%	34.76	Best choice for precise M isolation in research and therapy.
Stimulant	M	High power, less selective	Very high M (60.83%) but with strong L activation	S ≈ 1.90%, L ≈ 34.06%	89.17	For dynamic training requiring robust M drive despite spillover.
Delta	M	Alternative high power	Strong M (51.26%) with significant L activation	S ≈ 2.75%, L ≈ 34.28%	87.99	Consider in combined protocols with strict control of duration and luminance.
Theta	M	Moderate-high power	M = 47.12% with relevant L	S ≈ 1.02%, L ≈ 34.30%	86.57	Useful in intermediate phases where some spillover is acceptable.

Rationale percentages indicate modelled relative cone excitation, while transmittance (%), measured optical transmittance. Cross-talk entries indicate non-target cone activation. T (%) = Spectral transmittance at peak wavelength; S-cone = short-wavelength cone; M-cone = medium-wavelength cone; L-cone = long-wavelength cone.

**Table 5:** Spectral properties and cone-selectivity of L-cone–targeting filters.

Filter	Cone	Role	Rationale	Cross-Talk	T (%)	Clinical Note
Alpha	L	Maximum selectivity	Isolates L (8.50%) with negligible S and M	S ≈ 0.22%, M ≈ 0.01%	81.80	Best when selective L stimulation is required despite moderate amplitude.
Stimulant	L	High power	L = 34.06% but with strong M activation	S ≈ 1.90%, M ≈ 60.83%	89.17	For protocols prioritizing robust L activation under strict control.
Delta	L	Alternative strong option	L = 34.28% with high M contamination	S ≈ 2.75%, L ≈ 51.26%	87.99	To be used in short, high-intensity sessions with control.
Theta	L	Moderate-high power	L = 34.30% with relevant M	S ≈ 1.02%, L ≈ 47.12%	86.57	Appropriate for intermediate training phases with balanced stimulation.

Rationale percentages indicate modelled relative cone excitation, while transmittance (%), measured optical transmittance. Cross-talk entries indicate non-target cone activation. T (%) = Spectral transmittance at peak wavelength; S-cone = short-wavelength cone; M-cone = medium-wavelength cone; L-cone = long-wavelength cone.

**Table 6:** Filter spectral transmittance and cone-response characteristics of combined filters.

Combined Filter	Cone (Target)	Role	Rationale (S/M/L %)	Cross-Talk	Clinical Note
Upsilon– Neurasthenic	S	Selective S-combined	S = 9.72/ M = 0.01/ L = 0.02	M ≈ 0.01, L ≈ 0.02 (near-null)	Useful when an attenuated S stimulus is desired with minimal contamination; useful as a sensitization/diagnostic block or gentle S-bias start.
Omega–Pi	S	Selective S-combined (alt.)	S = 9.39/ M = 0.02/ L = 0.01	M ≈ 0.02, L ≈ 0.01 (near-null)	Alternative S-combined option when you want S isolation with very low spillover but accept lower absolute drive vs monochromatic filters.
Mu–Delta	M	Selective M-combined	S = 0.07/ M = 6.40/ L = 0.01	S ≈ 0.07 (very low), L ≈ 0.01 (near null)	M-centric combined block with excellent selectivity; suitable when you want M > 0 without dragging L.
Mu–Theta	M	Selective M-combined (alt.)	S = 0.03/ M = 5.22/ L = 0.01	S ≈ 0.03 (near-null), L ≈ 0.01 (near-null)	Interchangeable with Mu–Delta; pick based on patient tolerance or sequence design; preserves M emphasis with minimal spillover.
Delta–Theta	M/L (non-selective)	High-drive long-band combined	S = 0.10/ M = 36.87/ L = 30.58	High cross-talk (M & L both high)	Reserved for brief, controlled “push” blocks when robust long-band activation is justified, not for isolation due to cross-talk.
Alpha–Delta	L	Selective L-combined	S ≈ 0.00/ M = 0.01/ L = 7.70	S ≈ 0, M ≈ 0.01 (near-null)	L-centric combined option that keeps S/M near zero; useful when you want L bias with more control than mono high-power reds.

Rationale percentages indicate modelled relative cone excitation, while transmittance (%), measured optical transmittance. Cross-talk entries indicate non-target cone activation. T (%) = Spectral transmittance at peak wavelength; S-cone = short-wavelength cone; M-cone = medium-wavelength cone; L-cone = long-wavelength cone.

**Table 7:** Mechanistic mapping of S-, M-, and L-cone contributions across clinical conditions.

Condition	Cone Emphasis	Mechanistic Rationale
Amblyopia	L > M > S	L: parvocellular/foveal acuity deficits [77,78,86]. M: binocular integration/fusion. S: modulation via arousal/non-visual pathways [79].
Esotropia	S > M	S: ipRGC→OPN/EW parasympathetic–vergence coupling [83,84]. M: dorsal-stream fusional support [33,88]. L: minimized to reduce accommodative overdrive [71].
Exotropia	L > M (+ limited S)	L: parvocellular/foveal fixation stability [91]. M: dorsal-stream vergence integration [33,88]. S: limited arousal facilitation via ipRGC pathways [82].
Hypertropia	M > S	M: visuomotor/vergence timing via dorsal-stream inputs [33,88]. S: orienting/arousal via superior colliculus [92,93,94].
Hypotropia	L > M (+ limited S)	L: foveal/parvocellular fixation support [14,65]. M: visuomotor calibration [92]. S: ipRGC–pulvinar–SC orienting modulation [7,82].
ADHD	S + M + L combined	S: circadian/arousal regulation via ipRGC–SCN [7,101,102]. M: visuomotor/oculomotor tracking [33,88,97]. L: foveal/parvocellular fixation for sustained attention [18].

ipRGC = intrinsically photosensitive retinal ganglion cell; OPN = olivary pretectal nucleus; EW = Edinger–Westphal nucleus; SC = superior colliculus; SCN = suprachiasmatic nucleus; LGN = lateral geniculate nucleus.

**Table 8:** Condition-specific treatment duration frameworks for cone-targeted neuromodulation derived from prior studies and external literature. These values represent methodological anchors, for further therapeutic use.

Condition	Baseline Framework (Sessions)	Extended Framework (Weeks)	Evidence Base
Amblyopia	20 sessions [1,2]	12–16 weeks; sustained gains up to 1 year [80,81,108]	RCTs and digital/binocular therapy trials; classic patching often several months [77]
Strabismus	20 sessions [1,2]	8–12 weeks typical for orthoptic/binocular programs [89,90]	Cochrane reviews; no universal standard; other subtypes (eso-, hyper-, hypo-) remain investigational
ADHD	2–3 weeks baseline [101]	Up to 10 weeks in pilot/prevention RCTs [101,109,110,111]	Open trials and pilot RCTs of bright-light therapy; short protocols most common

RCT = randomized controlled trial; ADHD = attention-deficit/hyperactivity disorder.

**Table 9:** Clinical translation of cone-specific neuromodulation: integrated framework and condition-specific protocols.

Condition	Cone Priority	Key Isolation Filters	Key Combined Options	Session Outline (per Cone)	Safety/Age/Distance	Total Framework (Sessions/Weeks)
Amblyopia	L > M > S	Alpha (L), Mu (M), Omega (S)	Alpha–Delta; Mu–Delta/Mu–Theta; Upsilon–Neurasthenic/ Omega–Pi	S: 2 min × 4; 250–350 lx (morning/early day); M: 4 min × 4; 400–500 lx; L: 5 min × 4; 500–650 lx	Use diffuser; children 40–50 cm; adults 50–60 cm; elderly 30–40 cm; shorter blocks for <8 years	≈20 sessions baseline; extendable to 12–16 weeks
Strabismus (Esotropia)	S > M; avoid L	Omega (S), Mu (M)	Upsilon–Neurasthenic/ Omega–Pi; Mu–Delta/Mu–Theta	S: 2 min × 4; 250–350 lx (morning/early day); M: 4 min × 4; 400–500 lx; L avoided	Avoid glare; diffuser mandatory; children 40–50 cm; adults 50–60 cm; elderly 30–40 cm	≈20 sessions baseline; extendable up to 8–12 weeks
Strabismus (Exotropia)	L > M (+S brief)	Alpha (L), Mu (M), Omega (S brief)	Alpha–Delta; Mu–Delta/Mu–Theta; Upsilon–Neurasthenic/ Omega–Pi	L: 5 min × 4; 500–650 lx; M: 4 min × 4; 400–500 lx; S: 2 min × 4; 250–350 lx (brief, morning)	Diffuser use recommended; S brief blocks only; children 40–50 cm; adults 50–60 cm; elderly 30–40 cm	≈20 sessions baseline; extendable up to 8–12 weeks
ADHD	Integrated S + M + L	Omega (S), Mu (M), Alpha (L))	Upsilon–Neurasthenic/ Omega–Pi; Mu–Delta/Mu–Theta; Alpha–Delta; Delta–Theta	If run sequentially, use S→M→L to align with the timing windows in Table 2; total ≈ 44 min (8 + 16 + 20).; DC or PWM ≥ 1 kHz	S in morning only; children 40–50 cm; adults 50–60 cm; elderly 30–40 cm; shorter blocks for <8 years	2–3 weeks baseline; extendable up to 10 weeks

lx = lux (corneal illuminance); nm = nanometer (wavelength); DC = direct current; PWM = pulse width modulation; kHz = kilohertz; S-cone = short-wavelength cone; M-cone = medium-wavelength cone; L-cone = long-wavelength cone; Isolation filters = most selective for target cone; Moderate/High-drive filters = stronger stimulation with more cross-talk; Combined options = paired filters to reduce cross-talk or bias stimulation; Morning = early day session; Daytime = mid-day; Blocks = repeated intervals. Note: for all conditions: avoid 15–25 Hz; optional 32–40 Hz (in controlled research paradigm–not clinical use).

**Table 10:** Clinical protocol for amblyopia: operational parameters for L- and M-cone stimulation with optional S-cone input.

Cone	Isolation Filters	Moderate/High Drive Filters	Combined Options	Light Parameters	Safety/Age/Distance
L (primary)	Alpha	Stimulant/Delta/ Theta	Alpha–Delta	570 ± 15 nm; 500–650 lx; 5 min × 4 (≈20 min); daytime; DC or PWM ≥ 1 kHz	Daytime; shorter blocks for children <8 years; children 40–50 cm; adults 50–60 cm; elderly 30–40 cm
M (support)	Mu	Stimulant/Delta/ Theta	Mu–Delta/ Mu–Theta	530 ± 15 nm; 400–500 lx; 4 min × 4 (≈16 min); daytime; DC or PWM ≥ 1 kHz	Gradual ramps; caution in migraine prone; children 40–50 cm; adults 50–60 cm; elderly 30–40 cm
S (optional)	Omega	Lambda/Upsilon (moderate); Pi/Depressant (high drive)	Upsilon–Neurasthenic/ Omega–Pi	440 ± 15 nm; 250–350 lx; 2 min × 4 (≈8 min); morning/early day; DC or PWM ≥ 1 kHz	Caution in epilepsy/ photosensitivity; use diffuser; slow ramps

lx = lux; nm = nanometer; DC = direct current; PWM = pulse width modulation; kHz = kilohertz; S = short-wavelength cone; M = medium-wavelength cone; L = long-wavelength cone; gradual ramp = progressive increase in light intensity for comfort; Isolation filters = most selective for target cone; Moderate/High-drive filters = stronger stimulation with more cross-talk; Combined options = paired filters to reduce cross-talk or bias stimulation; Morning = early day session; Daytime = mid-day; Blocks = repeated intervals.

**Table 11:** Protocol framework for esotropia: S-cone–driven approach with avoidance of L-cone stimulation.

Cone	Isolation Filters	Moderate/High Drive Filters	Combined Options	Light Parameters	Safety/Age/Distance
S (primary)	Omega	Lambda/Upsilon (moderate); Pi/Depressant (high drive)	Upsilon–Neurasthenic/ Omega–Pi	440 ± 15 nm; 250–350 lx; 2 min × 4 (≈8 min); morning/early day; DC or PWM ≥ 1 kHz	Children 40–50 cm; adults 50–60 cm; elderly 30–40 cm; caution in epilepsy/ photosensitivity; diffuser required
M (secondary)	Mu	Stimulant/Delta/ Theta	Mu–Delta/ Mu–Theta	530 ± 15 nm; 400–500 lx; 4 min × 4 (≈16 min); daytime; DC or PWM ≥ 1 kHz	Gradual ramps; avoid glare; children 40–50 cm; adults 50–60 cm; elderly 30–40 cm
L	—	—	Avoid	—	Contraindicated (risk of over accommodation and worsening esodeviation)

lx = lux; nm = nanometer; DC = direct current; PWM = pulse width modulation; kHz = kilohertz; S = short-wavelength cone; M = medium-wavelength cone; L = long-wavelength cone; gradual ramp = progressive increase in light intensity for comfort; (—) = not applicable or generally avoided/minimized (risk of accommodative overdrive); Isolation filters = most selective for target cone; Moderate/High-drive filters = stronger stimulation with more cross-talk; Combined options = paired filters to reduce cross-talk or bias stimulation; Morning = early day session; Daytime = mid-day; Blocks = repeated intervals.

**Table 12:** Clinical protocol for exotropia: L-cone emphasis with M-cone support and restricted S-cone input.

Cone	Isolation Filters	Moderate/High Drive Filters	Combined Options	Light Parameters	Safety/Age/Distance
L (primary)	Alpha	Stimulant/Delta/ Theta	Alpha–Delta	570 ± 15 nm; 500–650 lx; 5 min × 4 (≈20 min); daytime; DC or PWM ≥ 1 kHz	Use diffuser if glare; children 40–50 cm; adults 50–60 cm; elderly 30–40 cm
M (secondary)	Mu	Stimulant/Delta/ Theta	Mu–Delta/ Mu–Theta	530 ± 15 nm; 400–500 lx; 4 min × 4 (≈16 min); daytime; DC or PWM ≈ 1 kHz	Gradual ramps; caution in photophobia; children 40–50 cm; adults 50–60 cm; elderly 30–40 cm
S (brief optional)	Omega	Lambda/Upsilon (moderate); Pi/Depressant (high-drive)	Upsilon–Neurasthenic/ Omega–Pi	440 ± 15 nm; 250–350 lx; 2 min × 4 (≈8 min); morning/early day; DC or PWM ≥ 1 kHz	Brief morning blocks only; caution in epilepsy/migraine; diffuser recommended

lx = lux; nm = nanometer; DC = direct current; PWM = pulse width modulation; kHz = kilohertz; S = short-wavelength cone; M = medium-wavelength cone; L = long-wavelength cone; gradual ramp = progressive increase in light intensity for comfort; Isolation filters = most selective for target cone; Moderate/High-drive filters = stronger stimulation with more cross-talk; Combined options = paired filters to reduce cross-talk or bias stimulation; Morning = early day session; Daytime = mid-day; Blocks = repeated intervals.

**Table 13:** Clinical protocol for ADHD: sequential S→M→L stimulation with combined-filter flexibility.

Cone	Isolation Filters	Moderate/High Drive Filters	Combined Options	Light Parameters	Safety/Age/Distance
S (arousal)	Omega	Lambda/Upsilon (moderate); Pi/Depressant (high-drive)	Upsilon–Neurasthenic/ Omega–Pi	440 ± 15 nm; 250–350 lx; 2 min × 4 (≈8 min); morning/early day; DC or PWM ≥ 1 kHz	Morning/early day; avoid late evening; children 40–50 cm; adults 50–60 cm; elderly 30–40 cm; caution in epilepsy/ photosensitivity; diffuser recommended
M (visuomotor)	Mu	Stimulant/Delta/ Theta	Mu–Delta/ Mu–Theta	530 ± 15 nm; 400–500 lx; 4 min × 4 (≈16 min); daytime; DC or PWM ≥ 1 kHz	Gradual ramps; observe oculomotor stability; children 40–50 cm; adults 50–60 cm; elderly 30–40 cm
L (fixation)	Alpha	Stimulant/Delta/ Theta	Alpha–Delta	570 ± 15 nm; 500–650 lx; 5 min × 4 (≈20 min); daytime; DC or PWM ≥ 1 kHz	Adjust for glare; avoid evening; children 40–50 cm; adults 50–60 cm; elderly 30–40 cm
Multi-bias combined	—	—	S-bias: Upsilon–Neurasthenic/ Omega–Pi; M-bias: Mu–Delta/Mu–Theta; L-bias: Alpha–Delta; M + L: Delta–Theta	Sequential S→M→L ≈ 44 min total: (8 + 16 + 20); DC or PWM ≥ 1 kHz	Age-adapt dosing; shorter blocks for children < 8 years

lx = lux; nm = nanometer; DC = direct current; PWM = pulse width modulation; kHz = kilohertz; S = short-wavelength cone; M = medium-wavelength cone; L = long-wavelength cone; ADHD = attention-deficit/hyperactivity disorder; sequential order S→M→L = short-wavelength (S) in morning, followed by medium- (M) and long-wavelength (L) stimulation during daytime; gradual ramp = progressive increase in light intensity for comfort; (—) = not applicable or generally avoided/minimized; Isolation filters = most selective for target cone; Moderate/High-drive filters = stronger stimulation with more cross-talk; Combined options = paired filters to reduce cross-talk or bias stimulation; Morning = early day session; Daytime = mid-day; Blocks = repeated intervals.

## Data Availability

The data presented in this study are available on request from the corresponding author. The data are not publicly available due to institutional data governance policies of the data-providing center.

## List of Abbreviations

ADHD	attention-deficit/hyperactivity disorder
ASD	autism spectrum disorder
α-opic EDI	equivalent daylight illuminance using CIE S 026 receptor weightings
CCT	correlated colour temperature
cd/m^2^	candela per square meter (luminance)
CIE	Commission Internationale de l’Éclairage
	(International Commission on Illumination)
CRI	colour rendering index
DC	direct current (steady illumination)
EDI	equivalent daylight illuminance
EEG	electroencephalography
EN	European Norm (standards body)
EW	Edinger–Westphal nucleus
fMRI	functional magnetic resonance imaging
HCs	healthy controls
Hz	hertz
ICNIRP	International Commission on Non-Ionizing Radiation Protection
IEC	International Electrotechnical Commission
IEEE	Institute of Electrical and Electronics Engineers
INC	interstitial nucleus of Cajal
ipRGCs	intrinsically photosensitive retinal ganglion cells
IRB	Institutional Review Board
ISO	International Organization for Standardization
kHz	kilohertz
L-cone	long-wavelength cone photoreceptor
LED	light-emitting diode
LGN	lateral geniculate nucleus
L > M > S	shorthand notation for cone weight emphasis
lx	photopic illuminance at the corneal plane (lux)
M-cone	medium-wavelength cone photoreceptor
min	minute
MST	medial superior temporal area
MT/V5	middle temporal visual area
N75, P100	visual evoked potential components (negative at 75 ms, positive at 100 ms)
NIR	near-infrared
nm	nanometer
NOEL	no-observable-effect level
OPN	olivary pretectal nucleus
PWM	pulse-width modulation
qEEG	quantitative electroencephalography
RCT	randomized controlled trial
RGB	red–green–blue primaries
RGW	red–green–white primaries
riMLF	rostral interstitial medial longitudinal fasciculus
SA	strabismus and amblyopia
S-cone	short-wavelength cone photoreceptor
SC	superior colliculus
SCN	suprachiasmatic nucleus
s	second
SPD	spectral power distribution
ssVEP	steady-state visual evoked potential
t½	half-time (adaptation constant)
UV	ultraviolet
UV–VIS–NIR	ultraviolet–visible–near-infrared spectrum
VA	visual acuity
V1, V2, V4	primary and extrastriate visual cortical areas
VEP	visual evoked potential
VF	visual field
VIS	visible spectrum
µm	micrometer
VR	virtual reality
